# Acute advanced aortic stenosis

**DOI:** 10.1007/s10741-023-10312-7

**Published:** 2023-04-21

**Authors:** Marisa Avvedimento, Domenico Angellotti, Federica Ilardi, Attilio Leone, Maria Scalamogna, Domenico Simone Castiello, Rachele Manzo, Andrea Mariani, Maddalena Immobile Molaro, Fiorenzo Simonetti, Carmen Anna Maria Spaccarotella, Raffaele Piccolo, Giovanni Esposito, Anna Franzone

**Affiliations:** Department of Advanced Biomedical Sciences, Federico II University of Naples, Via S. Pansini, 5 - 8031 Naples, Italy

**Keywords:** Aortic stenosis, Heart failure, Transcatheter aortic valve replacement, Decompensation, Urgent, Balloon aortic valvuloplasty

## Abstract

Acute decompensation often represents the onset of symptoms associated with severe degenerative aortic stenosis (AS) and usually complicates the clinical course of the disease with a dismal impact on survival and quality of life. Several factors may derange the faint balance between left ventricular preload and afterload and precipitate the occurrence of symptoms and signs of acute heart failure (HF). A standardized approach for the management of this condition is currently lacking. Medical therapy finds very limited application in this setting, as drugs usually indicated for the control of acute HF might worsen hemodynamics in the presence of AS. Urgent aortic valve replacement is usually performed by transcatheter than surgical approach whereas, over the last decades, percutaneous balloon valvuloplasty gained renewed space as bridge to definitive therapy. This review focuses on the pathophysiological aspects of acute advanced AS and summarizes current evidence on its management.

## Introduction

Degenerative aortic stenosis (AS) is the most frequent heart valve disease in industrialized countries with an increasing prevalence due to population aging [1]. Over the last decades, the widespread adoption of minimally invasive procedures to treat severe AS has raised a better awareness of the disease and significantly widened the number of patients who can benefit from safe and effective intervention [2]. Nevertheless, a relevant proportion of AS patients is referred to treatment when acute decompensation occurs with potential dismal impact on clinical outcomes. Heart failure (HF) features subjects with severe AS and is recognized as ultimate cause of death, regardless of treatment modality [3, 4]. In particular, an excess of mortality risk has been reported among patients with history of recent hospitalization for acute HF prior to transcatheter aortic valve replacement (TAVR) [5]. In addition, fixing AS, by either surgical or transcatheter valve replacement, does not reset the risk of recurrent episodes of HF [6].

This review summarizes the main pathophysiologic concepts of acute advanced AS and provides an updated overview of the current available tools to manage this condition. For this purpose, we included evidence from randomized clinical trials, meta-analysis, and prospective or retrospective cohort studies, identified by a systematic search in PubMed, Medline, and Embase from each database inception to June 2022. Case reports and letters were excluded, as well as non-English literature.

## Definition and prevalence of acute advanced AS

A standardized definition of acute advanced AS is currently lacking. Thus, ascertaining the actual prevalence of this condition is primarily challenged by the heterogeneity of clinical presentations reported across the studies. In addition, variability is also related to the type of analyzed cohorts that may include candidates to surgical aortic valve replacement (SAVR), percutaneous balloon aortic valvuloplasty (BAV) or TAVR. In most of cases, acute decompensation coincides with the need for unplanned hospitalization and/or urgent intervention and has been reported in up to 20% of patients with severe AS [7]. Acute events are reported both in patients not aware of their valve disease and in those with an established diagnosis and undergoing periodic monitoring [7]. Typical signs and symptoms of HF are consistently reported and include dyspnea (categorized as NYHA functional class ≥ 3), pulmonary edema, need for intravenous diuretic administration, paroxysmal nocturnal dyspnea, lower limb edema, X-ray signs of pulmonary congestion, pulmonary rales, jugular vein distension, and hepatomegaly. The prevalence of these conditions ranges from 9 to 60% [8–12].

Moreover, the spectrum of clinical presentation may also include patients with pre-shock or cardiogenic shock [13–15]. Clinical features that enhance the risk for acute decompensation include advanced age, female sex, history of coronary artery disease (CAD), prior episodes of congestive HF, anemia, combined valve disorders, atrial fibrillation, frailty, chronic kidney disease, and oxygen-dependent lung disease [12, 15, 16]. Left ventricular ejection fraction (LVEF) is usually lower in patients with decompensate than stable AS; however, acute decompensation may occur regardless of LVEF as, especially in the setting of severe AS, this can be preserved despite an impaired cardiac function.

A low-flow, low-gradient severe AS may be observed in up to 10% of patients with severe AS. A low flow state may occur with reduced LVEF (classical low flow AS) or with preserved LVEF (paradoxical low flow), and it is often associated with a low transvalvular gradient given that the gradient is highly flow dependent. The low-flow condition is predominantly a result of pronounced LV concentric remodeling with advanced diastolic dysfunction, impaired LV filling, and reduced longitudinal systolic function. Patients with severe LV dysfunction and low cardiac output usually present relatively low transvalvular gradients leading to an underestimation of AS severity. This scenario poses some challenges in the assessment of severity and management of valvular disease, especially in the acute setting. When applicable, multimodality imaging based on dobutamine stress echocardiography allows risk stratification and clinical decision-making in patients with low-flow AS. Also, valve calcium quantification by CT may be helpful for the management of these patients, especially in those with no LV flow reserve in whom dobutamine stress echocardiography is inconclusive.

## Pathophysiology

Symptoms onset in patients with AS largely depends on the structural and functional changes of the left ventricle (LV) as response to long-standing pressure overload. Concentric hypertrophy allows for the preservation of a normal cardiac output despite an increased wall stress; however, at longer term, increased myocyte volume and exaggerated extracellular matrix with development of both diffuse interstitial and focal replacement fibrosis, are responsible for progressive left and right ventricular dysfunction [17]. During the latent phase of the disease, a near-normal cardiac output is maintained through a delicate balance between the preload (left ventricular end-diastolic pressure) that is elevated to provide an adequate stroke volume and the afterload (systemic vascular resistances) that is also increased to preserve perfusion pressures. Factors affecting preload or afterload, even marginally, may precipitate acute decompensation. By impairing ventricular compliance, cardiac hypertrophy shifts the left ventricle pressure–volume curve to the right; the resultant noncompliant ventricle is highly dependent on the atrial contribution for adequate filling. Conditions precipitating advanced AS include loss of diastolic filling by atrial systole with atrial fibrillation or atrioventricular dyssynchrony; shortened diastolic filling period in the case of tachycardia; decreased forward cardiac output with bradycardia; impaired LV relaxation because of acute myocardial ischemia. Similarly, abrupt volume shifts associated with relevant blood loss or dehydration may compromise cardiovascular reserve [18]. Moreover, other conditions, such as myocardial ischemia and moderate to severe mitral or tricuspid valve disease, can coexist with AS and contribute to progressive cardiac dysfunction.

## Treatment

Treatment of advanced AS is challenging and burdened by high rates of early and late mortality [19]. Time from symptoms onset to treatment is a critical determinant of prognosis [20]. Hemodynamic stabilization can be achieved by drugs and/or percutaneous BAV. However, prompt valve replacement is the only definitive therapy. Studies including patients undergoing SAVR invariably reported worse clinical outcomes after urgent than elective interventions, that also account for the smallest amount of overall procedures [21, 22]. On the contrary, minimally invasive nature and progressive streamlining of TAVR significantly expanded the range of therapeutic options for AS patients presenting with acute decompensation.

## Medical therapy

Drug use in the setting of severe AS is hampered by the presence of a fixed afterload and the need for maintaining a constant preload. Administration of loop diuretics is the mainstay of therapy in the setting of acute HF. Their effect consists of improving fluid retention and mitigating pulmonary congestion; however, in patients with severe AS, this action is blunted by the relatively fixed outflow obstruction. Moreover, they cause an intravascular volume depletion that reduces preload and cardiac output causing arterial hypotension and peripheral hypoperfusion.

Among other measures targeting fluid retention, tolvaptan, an oral selective V2 receptor antagonist, promoting free water excretion in the urine without affecting hemodynamics and renal function, proved safety and effectiveness in small pilot studies including subjects with acute advanced AS [23]. Vasodilators may relief dyspnea but caution is needed as they can reduce blood pressure and coronary perfusion. Although these agents have been traditionally avoided in patients with AS, a small study in 25 critically ill patients with severe AS and reduced EF, showed that, by preferentially decreasing afterload, intravenous nitroprusside was associated with an increase in cardiac index and stroke volume and reduction of pulmonary capillary wedge pressure and systemic vascular resistances without causing hypotension. However, the study did not include patients with mean arterial systolic pressure lower than 60 mmHg or under inotropic treatment [24].

Nitroglycerin is generally contraindicated in patients with severe AS because of the associated risk of marked hypotension. This effect did not emerge from a retrospective comparison of patients with or without AS presenting with acute pulmonary edema and treated with sublingual or intravenous nitroglycerin [25]. Overall, to date, there is not enough evidence supporting the safety and effectiveness of these agents in acute advanced AS.

Vasopressors and inotropes improve tissue perfusion and are usually indicate in the setting of cardiogenic shock. However, in the context of AS, the conveyed risk of major arrythmias as well as the eventual worsening of myocardial ischemia because of increased myocardial oxygen consumption, should be taken into specific account. There is limited experience with the use of levosimendan, a calcium sensitizing agent with inotropic and vasodilator action that does not affect oxygen requirements. Its use was associated with improved hemodynamics and cardiac function among patients with acute HF and severe AS with reduced EF [26].

## Percutaneous BAV

Percutaneous BAV was first described by Cribier in 1986 as an alternative to SAVR in elderly and/or high-risk patients with symptomatic AS [27]. Later, the same author described its emergent use for patients presenting with cardiogenic shock, resulting in significant improvement in cardiac index and transvalvular gradients [28]. Despite this early enthusiasm, an unfavorable prognostic impact related to early restenosis, high in-hospital, and 1-year mortality rapidly limited its use [29]. However, a renewed interest in the procedure accompanied the advent of TAVR while remarkable technical advances mitigated the risk of complications [30]. European and US guidelines recommend BAV as a bridge to further interventions in hemodynamically unstable patients [31, 32]. It can also be offered as stand-alone option to patients with limited life expectancy or unsuitable for TAVR or as temporary measure in the setting of acute advanced AS [33, 34]. An overview of studies reporting outcomes of BAV is reported in Table 1. Overall, acute procedural success, measured as reduction of at least 50% of transaortic pressure gradient, was consistently reported; however, early and mid-term outcomes remained poor, with more than half of patients dead at 1-year, with a steady trend over time [20]. Specifically, excess risk of mortality was observed when BAV was not followed by definitive therapy, if the delay to SAVR or TAVR was long or in patients requiring repeat procedure [14, 30, 35–38].

**Table 1:** Main characteristics and results of studies on percutaneous balloon aortic valvuloplasty

**First author, year**	*n*	**Male, %**	**Age (years)**	**STS score (%)**	**Mean gradient pre-BAV (mmHg)**	**Mean gradient post- BAV (mmHg)**	**Follow-up**	**Death, %**	**Stroke, %**	**Major vascular complications, %**
**Ben-Dor, 2010** [62]	262	44.7	81.7 ± 9.8	13.3 ± 6.7	42.0 ± 13.9	31.9 ± 11.8	181 days	50	1.9	6.9
**Saia, 2013** [30]	415	44.3	77.5 ± 10.9	-	48.7 ± 18.1	31.7 ± 12.8	2-year	57.4	5.6	3.4
**Eltchaninoff, 2014** [38]	323	58.2	80.5 ± 9.9	-	44.4 ± 18.9	20.7 ± 11.0	5-year	78^a^	1.8	6.8
**Szerlip, 2017** [37]	100	-	80.6 ± 9.6	11.4 ± 7.1	26.1	-	1-year	41	1.3	3.9
**Alkhouli, 2017 **[63]	3168	51.1	82 ± 7.8	-	-	-	In-hospital	8.5	1.8	7.6
**Bongiovanni, 2018** [14]	118	55.9	81.3 ± 7.6	-	-	-	30-day	33	0	3.4
**Eugène, 2018** [36]	40	55	79 ± 9	26 ± 15	47 ± 15	32 ± 10	2-year	68	-	0
**Debry, 2018** [20]	44	75	77.3 ± 8.1	23.4 ± 11.6	39.0 ± 14.0	25.3 ± 11.0	1-year	70	0	0
**Bularga, 2020** [35]	167	52.1	80.0	-	42.0	32.0	1-year	43	1.2	0.6

*BAV* balloon aortic valvuloplasty, *STS* Society of Thoracic Surgeons

^a^For the group of patients that received TAVR after BAV; 5-year mortality rates were 96.7% and 60% for patients receiving medical therapy or SAVR, respectively

## TAVR

Unstable patients have been excluded from clinical studies investigating the role of TAVR. Nevertheless, since its introduction in the clinical practice, this procedure is being widely performed in urgent/emergency settings. To comprehensively evaluate evidence coming from studies published in this field, they can be grouped in 3 main categories: studies comparing urgent/emergent with elective TAVR; studies evaluating TAVR outcomes in patients presenting with acute HF or acute advanced AS; studies assessing TAVR in the context of cardiogenic shock. The main clinical and procedural characteristics of analyzed patients are reported in Table 2. Variable findings emerged from studies in the first group. Landes et al. found similar rates of periprocedural complications and early mortality between 27 and 342 patients undergoing urgent and elective TAVR, respectively [16]. Among 40,042 TAVR from the Society of Thoracic Surgeons/American College of Cardiology Transcatheter Valve (STS/ACC TVT) Registry, 3952 (9.7%) were classified as urgent/emergent and were performed in patients with a greater burden of comorbidities, presenting more often with combined aortic regurgitation and stenosis or degenerated surgical bioprostheses; they experienced higher rates of acute kidney injury, 30-day and 1-mortality than elective patients [12]. Similar outcomes were observed in another large cohort including more than 42,000 patients in which, however, a higher proportion of urgent TAVR was reported (24%) [11]. Emergent TAVR was required in 5.4% of patients from a multicenter Asian registry and did not emerge as predictor of mortality [39]. Bianco et al. performed a risk adjusted analysis of urgent versus elective TAVR in 1193 patients and found no significant survival disadvantage at 5-year in the urgent group, that accounted for 20% of total population [40].

**Table 2:** Main clinical and procedural characteristics of patients with acute decompensate aortic stenosis receiving transcatheter aortic valve replacement

**First author, year**	*n*	**Male,** *n*** (%)**	**Age (years)**	**STS score (%)**	**NYHA class IV,** *n*** (%)**	**Cardiogenic shock,** *n*** (%)**	**Femoral access,** *n*** (%)**	**Self-expandable valve,** *n*** (%)**	**Balloon-expandable valve,** *n*** (%)**	**TAVR in degenerated prosthesis,** *n*** (%)**	**MCS,** *n*** (%)**
**Landes, 2016** [16]	27	12 (44.5)	80.1 ± 9.7	9.7	17 (63)	0	26 (96.3)	22(81.5)	5(18.5)	0	0
**Frerker, 2016** [15]	27	12 (44.5)	78 ± 9			27 (100)	25 (92.5)	16 (59.3)	11 (40.7)	0	0
**Kolte, 2018** [12]	3952	2050 (51.8)	84	11.8	2087 (52.8)	97 (2.5)	2980 (75.4)	931 (23.6)	2984 (75.5)	202 (5.1)	47 (1.2)
**Abdelaziz, 2018** [8]	76	38 (50)	81.7 ± 6.8		76 (100)	0	64 (84.2)	32(42)	0	0	0
**Bongiovanni, 2018** [14]	23	19 (82.6)	76.0 ± 11.4		22 (95.7)	2 (8.7)	-	8 (34.8)	15 (65.2)	0	0
**Elbadawi, 2020** [11]	10,089	5017 (49.7)	81.0 ± 9.05				8196 (81.2)				348 (3.4)
**Patel, 2020** [41]	170	88 (51.8)	83.1 ± 7.6				164 (96.5)	16 (9.4)	143 (84.1)	0	0
**Huang, 2019 **[64]	31	23 (74.2)	73.1 ± 13.9			26 (83.9)		5 (16.1)	25 (80.6)		16 (51.6)
**Fraccaro, 2020** [13]	51	25 (49)	75.8 ± 12.9	19.2		51 (100)	45 (88.2)	30(58.8)	12 (23.5)	13 (25.5)	6 (11.7)
**Jalava, 2020 **[65]	210	99 (47.1)	80.7 ± 6.6	8.1			210 (100)				
**Enta, 2020** [39]	87	26 (29.8)	84.9 ± 7.0	13.7	77 (88.5)		72 (82.8)	5(5.8)	82 (94.2)		14 (16.1)
**Maidman, 2020** [42]	17	2 (11.7)	74.7 ± 8.9	22.3	15 (88.2)	9 (52.9)	17 (100)	1 (5.9)	16 (94.1)		2 (11.7)
**Bianco, 2021** [40]	247	131 (53)	82		148 (59.9)	3 (1.2)	203 (82.2)	144 (58.3)	103 (41.7)	29 (11.7)	
**Lux, 2021 **[66]	53	30 (56.6)	79				36 (67.9)			7 (13.2)	
**Kaewkes, 2021 **[67]	139	74 (53)	82.2 ± 9.6	6.7	132 (95%)		129 (93)	23 (16)	116 (84)		

*MCS* mechanical circulatory support, *NYHA* New York Heart Association, *STS* Society of Thoracic Surgeons, *TAVR* transcatheter aortic valve replacement

Studies focusing on clinical presentation rather than urgency of the procedure also provided inconsistent results. Acceptable rates of in-hospital and 1-year death were reported among 76 patients with acute advanced AS undergoing transfemoral TAVR [8]. In a multicenter cohort of patients presenting with severe acute dyspnea (NYHA functional class IV), cardiac resuscitation or mechanical circulatory support (MCS), rates of procedural and 30-day mortality were as high as 8.7% and 23.8%, respectively [14]. In the study by Patel et al., patients presenting with ventricular tachycardia, ventricular fibrillation, aborted sudden death or preoperative cardiac massage, ventilation, inotropes, intra-aortic balloon pump (IABP) or acute renal failure had poor prognosis at 30-day and 1-year after TAVR [41]. When exclusively focusing on patients with cardiogenic shock, TAVR was generally associated with acceptable outcomes in relation to the critical clinical status at presentation. Freker et al. reported a device success rate of 89%, a survival probability of 59% at 1-year and no increased rates of periprocedural complications in 27 patients [15]. In a multicenter European study of 51 patients with cardiogenic shock, device success was 94.1%, and 30-day and 1-year mortality were 11.8% and 25.7%, respectively [13]. Comparable performance in terms of 30-day mortality was reported by a study assessing TAVR or SAVR in shock patients [42]. Table 3 provides a summary of the main results of these studies. A meta-analysis including 14 studies reported higher incidence of 30-day, in-hospital, and 1-year mortality after emergent than elective TAVR [43]. Performing TAVR in the setting of acute clinical decompensation requires some deviations from standard organizational pathways. For example, pre-procedural CT scan to assess vascular access and aortic valve can be avoided to save time and limit contrast medium use; abbreviated interruption of eventual oral anticoagulant therapy as well as postponement of the treatment of concomitant conditions such as relevant CAD could be necessary. Procedural volume may play a role on outcomes: an analysis of more than 25,000 procedures showed a direct relationship between total hospital TAVR volumes and amount of urgent/emergent procedures with a relevant impact on the performance as significantly lower rates of in-hospital mortality, stroke, and other complications occurred in high-volume hospitals [44].

**Table 3:** Main clinical outcomes of patients with acute decompensate aortic stenosis receiving transcatheter aortic valve replacement

**First author, year**	*n*	**Device success,** *n*** (%)**	**Follow-up**	**Stroke,** *n*** (%)**	**Major bleeding,** *n*** (%)**	**Vascular complications,** *n*** (%)**	**Acute kidney injury,** *n*** (%)**	**Moderate/severe PVL,** *n*** (%)**	**Mortality at longest follow-up,** *n*** (%)**
**Landes, 2016** [16]	27	23 (85.2)	30-day	1 (3.7)	1 (3.7)	5 (18.5)	4 (14.8)	2 (9.5)	1 (3.7)
**Frerker, 2016** [15]	27	24 (88.9)	1-year	1 (3.7)	1 (3.7)	9 (33.3)	8 (29.6)	1 (3.7)	11 (40.7)
**Kolte, 2018** [12]	3952	3659 (92.6)	1-year	91 (2.3)	324 (8.3)	39 (1.0)	323 (8.2)	159 (4.0)	1185 (29)
**Abdelaziz, 2018** [8]	76		2-year	2 (2.6)		7 (9.2)	4 (5.3)		14 (18.4)
**Bongiovanni, 2018** [14]	23		30-day	2 (8.7)	1 (4.3)	5 (21.7)	3 (13)	1 (4.3)	5 (23.8)
**Elbadawi, 2020** [11]	10,089		In-hospital	274 (2.7)	3628 (36)	214 (2.1)	2827 (28)		565 (5.6)
**Patel, 2020** [41]	170		30-day	2 (1.2)		9 (5.3)	20 (11.8)		9 (5.3)
**Huang, 2019 **[64]	31		2-year	3 (10)	2 (6.7)	5 (16.7)	5 (16.7)	0	13 (41.9)
**Fraccaro, 2020** [13]	51		30-day	1 (2)	2 (3.9)	2 (3.9)	17 (33.4)	8 (15.6)	6 (11.8)
**Jalava, 2019 **[65]	210		30-day	8 (3.8)	56 (26.8)		26 (12.3)	7 (3.3)	10 (4.8)
**Enta, 2020** [39]	87	76 (87.4)	30-day	3 (3.4)	19 (21.8)	13 (14.9)	20 (22.9)	2 (2.4)	8 (9.2)
**Maidman, 2020** [42]	17		30-day	0			0		2 (11.7)
**Bianco, 2021** [40]	247		30-day	9 (3.6)			7 (2.8)	13 (5.2)	16 (6.5)
**Lux, 2021 **[66]	53		1-year	1 (1.9)			1 (1.9)		15 (28.3)
**Kaewkes, 2021 **[67]	139		30-day	5 (4)	9 (6)	8 (5.7)	5 (4)	46 (39)	11 (8)

*PVL:* paravalvular leak

## Proposed treatment algorithm

**Fig. 1:**
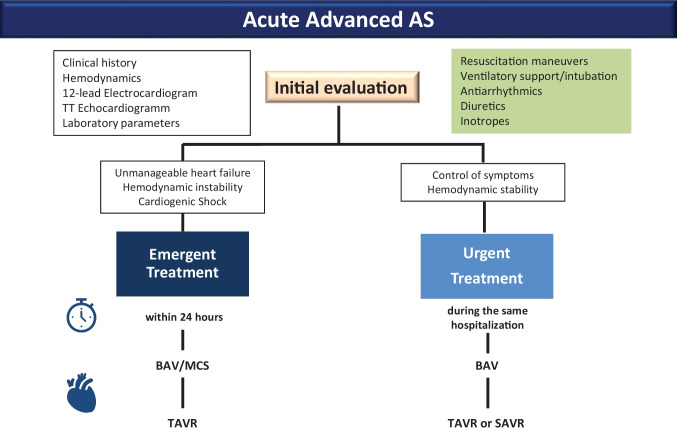
Management of patients with acute advanced aortic stenosis

In the absence of standardized protocols, the therapeutic strategy for patients presenting with acute advanced AS is now chosen based on local facilities and expertise with some centers pursuing hemodynamic stabilization by identification and control of precipitating factors and others opting for percutaneous BAV or urgent valve replacement. Nevertheless, based on available evidence, the following algorithm could be recommended (Fig. 1).

### Initial assessment

Initial evaluation should take into account respiratory status and drugs type and administration route at home, besides clinical history and risk factors. Prompt identification of new-onset rhythm disorders by standard 12-lead electrocardiogram is pivotal to target eventual triggers of decompensation. Echocardiography (even by point of care ultrasound), routine laboratory values, and eventual X-ray readily inform about LV function, filling pressures, pulmonary congestion, anemia, acute renal failure, and electrolytic imbalances, infections. The main goals at this stage are avoiding tachycardia, optimizing preload, and maintaining an adequate blood pressure. Resuscitation maneuvers, antiarrhythmic drugs, and ventilatory support might be needed. Intubation should be avoided, if possible, as it can aggravate hypotension. Diuretics can be used in case of volume overload (B-lines or signs of congestion), keeping in mind the need to avoid hypotension because of the close dependence of these patients from preload. Based on clinical and hemodynamic status at presentation as well as on the response to initial stabilization maneuvers, the definitive intervention can be classified either as emergent (to be performed without delay, within 24 h) or urgent (to be performed during the same hospitalization or within 7 days from admission). Treatment modality should be ideally discussed within the local heart team.

Concomitant coronary artery disease should also be considered before any therapeutic action on the valve, as it may impact procedural risk and patient prognosis [45]. In the STS/ACC TVT registry, a comparable prevalence of CAD and a slight increase of left main significant stenosis were reported among patients undergoing urgent TAVR as compared with those undergoing elective TAVR (63.3% vs 62.2% and 11.1 vs 10.0%, respectively) [7]. In the TAVI-shock registry, 8 of the 51 patients presenting with cardiogenic shock underwent percutaneous coronary intervention (PCI) in the same session of TAVR [8]. In the setting of acute advanced AS, invasive coronary angiography should be performed concomitant to TAVR to save time, and PCI of proximal significant lesions should be performed accordingly to guidelines. However, it should be considered that robust evidence for strong recommendations about coronary revascularization is lacking for urgent as well as for elective TAVR.

### Patients requiring urgent procedure

In this setting, usually, acceptable control of symptoms and hemodynamic stabilization can be achieved by medical therapy. In addition, percutaneous BAV is a reasonable option as bridge to valve replacement. It allows for a rapid relief of pressure overload that can have a dramatic impact on symptoms and hemodynamics and allows for a more accurate evaluation of the proper modality for valve replacement. BAV can be performed as palliative measure when general conditions prohibit any further intervention. Valve replacement by TAVR or SAVR should be then performed in the same hospitalization to minimize the risk of recurrences of severe symptoms.

### Management of patients requiring emergent treatment

Emergency is usually dictated by refractory or unmanageable HF with hemodynamic instability. A tailored approach is needed as the decision to intervene is also based on life expectancy, age, and comorbidities. Overall, in the acute scenario, TAVR should be preferred over SAVR because of its less invasiveness. In these cases, preprocedural assessment of valve anatomy for the prosthesis choice can be based on transesophageal echocardiography in place of CT-scan; prosthesis size selection can also be performed by BAV. Along the same line, suitability of femoral access can be assessed by ultrasound. Procedure should be carried out according to standard local protocol and all measures to minimize further hemodynamic instability or complications should be adopted. Specifically, in this setting, particular attention should be paid to avoid or shorten rapid pacing, to optimize valve positioning, minimize the risk of residual paravalvular leak and to ensure adequate hemostasis of vascular access. Careful monitoring of vital parameters and renal function in intensive care unit is recommended for at least 24–48 h thereafter.

In patients presenting with cardiogenic shock, extreme caution is needed when selecting the appropriate drugs. Fluid management is very challenging as patients may present with pulmonary congestion but are closely dependent from preload to maintain cardiac output and could not tolerate the volume depletion induced by diuretics. Extreme cases may require crystalloid infusion with continuous monitoring of volume status, particularly, to face the hypotension that can derive from intubation. Among vasopressors, phenylephrine (a pure alpha-1 agonist) increases diastolic blood pressure and maintains adequate coronary perfusion pressure; it can also cause reflex bradycardia with favorable impact on diastolic filling. Norepinephrine is a valid alternative. Epinephrine, on the contrary, should not be considered owing to its marked chronotropic effect that increases myocardial oxygen consumption. Low-dose inotropes are also useful, particularly in patients with low flow low gradient AS. Overall, catecholamines and inotropes should be given at the lowest dose and for limited duration.

Despite limited evidence supporting their use, escalation to devices for mechanical circulatory support (MCS) can be considered in cases of refractory shock. The purpose of MCS, such as intra-aortic balloon pump (IABP), Impella system (Abiomed, Inc., Danvers, Massachusetts), TandemHeart device (Cardiac Assist, Inc., Pittsburgh, PA) and Extra-Corporeal Membrane Oxygenation (ECMO), is to reduce LV work and myocardial oxygen demand while maintaining systemic and coronary perfusion, supporting a rapid hemodynamic stabilization. Several technical aspects must be considered when choosing a MCS device such as indications, access site, concomitant valvular disease, and operator experience [46]. Specifically, severe aortic insufficiency and right ventricular failure represents unfavorable conditions for MCS placement, since their use may increase afterload, predisposing to LV dilation, increased filling pressure, and pulmonary congestion. Also, IABP is contraindicated in aortic disease [47, 48]. Regarding technical feasibility, access remains a key issue. ECMO requires femoral artery and femoral vein cannulation, over wires which are already placed during transfemoral TAVR, while an intra-atrial trans-septal puncture is needed for TandemHeart placement, which may be time-consuming and challenging in emergency setting. To date, evidence on MCS adoption before or during TAVR procedure is limited. A systematic assessment of the performance of MCS in TAVR patients found that it was associated with a relevant increase in the risk of early mortality, total hospital cost and length of stay [49, 50]. It should be noted that these sobering findings might be attributable to the critical baseline conditions of patients in which their use represented, generally, a rescue measure. Moreover, recent evidence suggests that in patients undergoing TAVR and requiring MCS, the prophylactic MCS implantation is associated with a significantly lower periprocedural mortality compared to patients with a rescue insertion during the procedure [51]. These findings support the use of a planned strategy to improve clinical outcomes in very high-risk aortic interventions. Moreover, improved hemodynamics by MCS enhances the tolerability of further intervention such as BAV or TAVR. The latter should be preferred in this setting and performed without significant delay.

## Residual risk of acute decompensation after treatment

The majority of rehospitalizations for cardiovascular causes within the first year after TAVR occur because of HF [52, 53]. Predictive factors have been identified and include diabetes, chronic lung disease, prior episodes of acute HF, pulmonary hypertension and moderate or severe paravalvular leak [53, 54]. A dismal prognostic impact has also been reported with higher risk for subsequent mortality, especially in cases requiring multiple readmissions [6]. A propensity-matched cohort comparison of readmission rates after transcatheter treatment of approximately 12,000 patients with acute advanced AS found a significantly lower risk after urgent TAVR than BAV [55]. Nevertheless, there is an overall significant burden of rehospitalizations on healthcare economics and patients quality of life. Failure of reversal of the structural and functional myocardial changes induced by longstanding AS may explain the residual risk for HF after TAVR. The trajectory of LV mass regression largely varies among patients and fixing AS in advanced stages of disease can yield a null effect on cardiac function [56]. A comprehensive approach to mitigate the risk of irreversible alterations of cardiac structures is being advocated [57]. It includes two main components: the first is the identification of optimal timing to intervene and there is growing evidence that this should be anticipated to the asymptomatic phase of the disease or even when the stenosis is still moderate [58, 59]. The second is the systematic implementation of medical therapy targeting cardiac remodeling [60, 61].

## Conclusions

Multiple factors can precipitate acute decompensation in patients with severe AS. They represent an extremely high-risk population, with poor prognosis in the absence of a definitive treatment. The use of medical therapy is narrowed by an unfavorable risk to benefit ratio of drugs that can worsen hemodynamics of severe AS. Urgent SAVR is rarely performed because of the prohibitive operative risk. Among transcatheter-based therapies, BAV can palliate symptoms but should be considered as a temporary measure owing to the high rates of failure occurring at early and mid-term while TAVR is consolidating its role as effective and safe stand-alone option in this setting. Technical improvements are expected to further expand the use of TAVR in acute advanced AS. However, continuous efforts should converge on the identification of actions that can minimize the occurrence of critical clinical situations requiring urgent intervention. In this perspective, anticipating the treatment of AS prior to symptoms onset and at earlier stages of cardiac damage seems the way ahead.

## Data Availability

All data and materials underlying this article will be shared on reasonable request to the corresponding author.

## Abbreviations

AS: Aortic stenosis
BAV: Balloon aortic valvuloplasty
CAD: Coronary artery disease
EF: Ejection fraction
HF: Heart failure
IABP: Intra-aortic balloon pump
LV: Left ventricle
MCS: Mechanical circulatory support
PCI: Percutaneous coronary intervention
SAVR: Surgical aortic valve replacement
TAVR: Transcatheter aortic valve replacement
