# Correction to: Acute advanced aortic stenosis

**DOI:** 10.1007/s10741-023-10320-7

**Published:** 2023-07-01

**Authors:** Marisa Avvedimento, Domenico Angellotti, Federica Ilardi, Attilio Leone, Maria Scalamogna, Domenico Simone Castiello, Rachele Manzo, Andrea Mariani, Maddalena Immobile Molaro, Fiorenzo Simonetti, Carmen Anna Maria Spaccarotella, Raffaele Piccolo, Giovanni Esposito, Anna Franzone

**Affiliations:** Department of Advanced Biomedical Sciences, Federico II University of Naples, Via S. Pansini, 5 ‑ 8031 Naples, Italy

**Correction to: Heart Failure Reviews** **https://doi.org/10.1007/s10741-023-10312-7**

Unfortunately, there was an error in the name of one of the authors. It is with utmost sincerity that we inform you that Domenico Simone Catiello has been erroneously attributed to the author instead of Domenico Simone Castiello.

Additionally, Maddalena Immobile Molaro's name has been presented inaccurately. To rectify this, we wish to clarify that the author's surname is Immobile Molaro and her name is simply Maddalena, with no middle name.

Our sincerest apologies for any inconvenience caused by these mistakes.

The original article has been corrected.

